# P03 Improving antimicrobial stewardship and sustainability by promoting oral metronidazole in eligible surgical patients at the Royal Alexandra Hospital

**DOI:** 10.1093/jacamr/dlac053.003

**Published:** 2022-05-31

**Authors:** Rachael Rodger, Amy Robertson, Caroline McGee, Lindsey Chisholm, Helen Ballantyne, Claire McCutcheon

**Affiliations:** NHS Greater Glasgow & Clyde, UK

## Abstract

**Background:**

Antimicrobial stewardship (AS) is essential to minimize the emergence and spread of antimicrobial resistance.^1^ Timely IV to oral antibiotic switch and promotion of the oral route are key AS initiatives in the acute hospital setting.^1^ Metronidazole is commonly prescribed IV in the surgical management of intra-abdominal sepsis; however, metronidazole has excellent oral bioavailability of 95%–100%. In addition to improved AS, the oral route provides advantages to patients, staff and the environment including reduced patient risk of line infections/complications; reduced staff workload and healthcare costs; improved patient comfort/mobility; earlier discharge; and improved sustainability.

**Objectives:**

To measure baseline proportion of surgical patients meeting the criteria for oral receiving oral metronidazole; introduce tests of change to promote use of oral metronidazole where appropriate; and measure changes in prescribing behaviour to determine if improvement has been achieved, with the aim of increasing the proportion of oral metronidazole prescribed to eligible Royal Alexandra Hospital (RAH) surgical patients to 50% by February 2022.

**Methods:**

Baseline metronidazole prescribing data were collected over 6 weeks in surgical wards at the RAH. Exclusions for oral metronidazole included compromised gut absorption, oral route unreliable or worsening clinical condition/sepsis. A quality improvement approach was used to engage and raise awareness of the multidisciplinary surgical team to the benefits of prescribing oral metronidazole where appropriate. A range of methods were used including prospective audit and feedback; departmental presentations to surgical and pharmacy teams; eye-catching posters in key areas; staff champions; staff recognition awards; WhatsApp^©^ group messages; and West of Scotland Surgical Association presentation. To measure improvement the proportion of oral and IV metronidazole prescribed was measured weekly and oral and IV metronidazole usage data in RAH surgical wards was measured monthly. A questionnaire was used to assess the impact of the project on nursing staff.

**Results:**

Baseline data (*n* = 90) highlighted that only 13.5% of patients meeting the criteria for oral metronidazole were prescribed oral in RAH surgical wards. Post-change: oral metronidazole prescribing increased from a baseline median of 7% to 51%; IV metronidazole usage data decreased by 45%; there were 320 fewer IV metronidazole administrations per month; and nursing staff reported benefits including reduced workload, improved patient comfort and reduced plastic waste.

**Conclusions:**

A quality improvement approach to engage and raise staff awareness of the benefits of oral metronidazole resulted in a change in prescribing behaviour and increased the proportion of oral metronidazole prescribed to eligible surgical patients to 50% by February 2022.
